# Impact of partial versus whole breast radiation therapy on fatigue, perceived stress, quality of life and natural killer cell activity in women with breast cancer

**DOI:** 10.1186/1471-2407-12-251

**Published:** 2012-06-18

**Authors:** Kevin Albuquerque, Dina Tell, Philip Lobo, Linda Millbrandt, Herbert L Mathews, Linda Witek Janusek

**Affiliations:** 1 Department of Radiation Oncology, Loyola University Health System, Maywood, IL, USA; 2 Marcella Niehoff School of Nursing, Loyola University Chicago, Maywood, IL, USA; 3 Radiation Oncology, Northwest Community Hospital, Arlington Heights, IL, USA; 4 Department of Microbiology and Immunology, Stritch School of Medicine, Loyola University Chicago, Maywood, IL, USA; 5 Moncrief Radiation Oncology Center, UT Southwestern Medical Center, 5801 Forest Park Road, Dallas, USA

**Keywords:** Breast Radiation, Fatigue, Perceived Stress, Quality of Life, Natural Killer Cell Activity

## Abstract

**Introduction:**

This pilot study used a prospective longitudinal design to compare the effect of adjuvant whole breast radiation therapy (WBRT) versus partial breast radiation therapy (PBRT) on fatigue, perceived stress, quality of life and natural killer cell activity (NKCA) in women receiving radiation after breast cancer surgery.

**Methods:**

Women (N = 30) with early-stage breast cancer received either PBRT, Mammosite brachytherapy at dose of 34 Gy 10 fractions/5 days, (N = 15) or WBRT, 3-D conformal techniques at dose of 50 Gy +10 Gy Boost/30 fractions, (N = 15). Treatment was determined by the attending oncologist after discussion with the patient and the choice was based on tumor stage and clinical need. Women were assessed prior to initiation of radiation therapy and twice after completion of radiation therapy. At each assessment, blood was obtained for determination of NKCA and the following instruments were administered: Perceived Stress Scale (PSS), Functional Assessment of Cancer Therapy-Fatigue (FACT-F), and Functional Assessment of Cancer Therapy-General (FACT-G). Hierarchical linear modeling (HLM) was used to evaluate group differences in initial outcomes and change in outcomes over time.

**Results:**

Fatigue (FACT-F) levels, which were similar prior to radiation therapy, demonstrated a significant difference in trajectory. Women who received PBRT reported progressively lower fatigue; conversely fatigue worsened over time for women who received WBRT. No difference in perceived stress was observed between women who received PBRT or WBRT. Both groups of women reported similar levels of quality of life (FACT-G) prior to initiation of radiation therapy. However, HLM analysis revealed significant group differences in the trajectory of quality of life, such that women receiving PBRT exhibited a linear increase in quality of life over time after completion of radiation therapy; whereas women receiving WBRT showed a decreasing trajectory. NKCA was also similar between therapy groups but additional *post hoc* analysis revealed that better quality of life significantly predicted higher NKCA regardless of therapy.

**Conclusions:**

Compared to WBRT, PBRT results in more rapid recovery from cancer-related fatigue with improved restoration of quality of life after radiation therapy. Additionally, better quality of life predicts higher NKCA against tumor targets, emphasizing the importance of fostering quality of life for women undergoing adjuvant radiation therapy.

## Background

Radiation therapy after surgical removal of an early breast cancer is a very important part of breast conservation treatment. The classic radiation schedule consists of a course of whole breast radiation therapy (WBRT) lasting for 6–6.5 weeks, which targets the entire breast tissue and underlying structures. An alternative approach is partial breast radiation therapy (PBRT), using brachytherapy. This approach targets only the breast tissues around the tumor bed and is administered over a short-course of five days. Brachytherapy for breast cancer is an evolving technique that can simplify radiation therapy, reduce toxicity, increase patient convenience, and possibly increase utilization of breast-conserving approaches to treatment. Results from several phase I and II studies document that accelerated PBRT using interstitial catheters has produced excellent 5-year results with regard to local tumor control, toxicity, and cosmesis [1-4]. In 2009 the American Society of Radiation Oncology published guidelines and recommendations for the use of PBRT [5]. However, definitive outcomes regarding local control and survival await completion of an ongoing national study comparing PBRT to WBRT [5,6].

Cancer and its treatment result in behavioral symptom distress, and one of the most pervasive and distressing symptoms is fatigue [7]. Cancer-related fatigue is more intense than typical fatigue and may be due to the disease itself and/or cancer treatment [8]. Most women undergoing radiation therapy for breast cancer experience fatigue [9], which typically subsides within weeks after completion of radiation therapy [10]. Yet, for some women, fatigue persists well beyond cancer treatment [11]. A longitudinal study of women with breast cancer found that 35% of women reported fatigue 1–5 years after treatment [12], while 5–10 years later, 34% continued to experience fatigue [13]. Variation in fatigue trajectories may also be related to dose and field size of radiation [14], as well as psychological and personal factors [15]. The human cost of cancer-associated fatigue is high, as it may lead to interruption of or discontinuation of cancer treatment [16,17]. Fatigue is also associated with shorter recurrence-free survival and overall survival in women with breast cancer [18]. It is clear that fatigue impairs overall quality of life [8,19,20] and, if chronic, fatigue can increase the need for healthcare services and result in lost wages [21].

The biological mechanism(s) underlying radiation-associated fatigue are unclear; however, evolving evidence implicates alterations in immune effector function, namely increased proinflammatory mediators [9,22]. Other types of immune dysregulation have also been associated with cancer treatment, most notably, reduced natural killer cell activity (NKCA) [23-25]. Reduced NKCA may have important long term implications for cancer patients in that NK cells defend against tumor metastasis, tumor initiation, and primary tumor growth [26-29]. Epithelial tumors, such as breast cancer, are especially susceptible to the anti-tumor effects of NK cells [27,30-34]. During critical times marked by risk for tumor dissemination, such as after surgery and during the early phase after completion of adjuvant radiation therapy, NK cell mediated anti-tumor defense becomes particularly important [35-38].

Evidence also suggests that adjuvant breast radiation may directly alter immune function [39,40]. Given that WBRT involves radiation of a greater volume of breast and normal tissues, with tangential fields that includes the lower axillary nodes, it may produce greater alterations of immune function [41], contributing to greater fatigue [22]. In contrast, PBRT is a localized treatment and does not include lymph nodes in the radiation field. Yet, for PBRT the radiation dose is given rapidly and in larger fractions, which may have other associated toxicity risks [42].

To date, studies comparing WBRT to PBRT have primarily focused on ‘local control’ and ‘cosmesis,’ as emphasized by the National Surgical Breast and Bowel Project [5,6]. Few studies have evaluated these two types of radiation for other outcomes, such as behavioral symptom distress and quality of life [14,43-45]; while no studies to our knowledge have compared immune outcomes, like NKCA. Thus, the purpose of this study was to compare the effect of adjuvant WBRT versus PBRT on fatigue, perceived stress, quality of life and NKCA in women receiving adjuvant radiation therapy after breast cancer surgery.

## Methods

### Design and participants

This was a non-randomized prospective multi-site pilot study, fully approved by participating institutional review boards for the study of human subjects. Treatment was determined by the oncologist after discussion with the patient. Choice was based on tumor stage and clinical need. Participant eligibility criteria for the PBRT group were as follows: age >45 years; non-lobular histology and DCIS, pathological stages T1 (lesions ≤ 2 cm), N0 M0; unilateral breast cancer, negative surgical margins (> 2 mm) both for the invasive component and the DCIS component; no extensive intraductal component. For the WBRT group, to maintain homogeneity, criteria were the same except that lobular histologies were allowed. All grades of DCIS were eligible. Common additional exclusion criteria for both groups were as follows. Women were excluded if they: received chemotherapy, had recurrent breast cancer, had major immune-based disease or dysfunction, were diagnosed with psychoses, were drug or alcohol abusers, were taking corticosteroids, anxiolytics or antidepressant drugs, or drugs known to affect the immune system.

### Procedures

Following breast conserving surgery and prior to initiation of radiation treatment, the purpose and nature of the study was discussed and eligible women were invited to participate. Women were enrolled from Loyola University Medical Center and Northwest Community Hospital. After obtaining informed consent, fatigue, perceived stress, and quality of life were measured by self-report using established psychometric instruments. Demographic information and medical history were obtained by patient interview and medical record review. Blood was drawn between 9 AM-4 PM by venipuncture (30 ml) for NKCA and immediately transported to the research laboratory. Subsequent data collection occurred in the clinic setting. Outcome variables were measured prior to the initiation of any radiation therapy and at two time periods after completion of radiation therapy. Post radiotherapy time points were anchored with respect to the end of radiation therapy. The second time point for women in the WBRT group took place upon completion of the whole breast radiation therapy, approximately 7 weeks after the initial time point and for women in the PBRT upon completion of the partial radiation therapy, approximately 6 days after the initial time point. The third time point was 6 weeks after the T2 for both groups. The difference in the data collection schedules was accommodated by the statistical approach, which projected outcomes through 6-weeks after radiation treatment (discussed below).

### Radiation techniques

The technique of PBRT using Mammosite has been described elsewhere [45]. A CT scan was obtained for brachytherapy planning and verification films were taken at each fraction. The dose prescribed at 1 cm from the surface of the balloon was 3400 cGy in ten fractions twice a day over five days. WBRT was delivered with 3-dimensional conformal techniques with 50 Gy in 25 fractions followed by a 10 Gy lumpectomy bed boost in 5 fractions. The patients in the two groups were not directly matched, but demographics were similar due to the strict eligibility criteria.

### Behavioral measures

#### Functional assessment of cancer therapy – fatigue – (FACT-F)

FACT-F is a 13-item scale used extensively in individuals with cancer. FACT-F has good test-retest reliability (0.87) and strong internal consistency (coefficient alpha = 0.93). Choices for each item on the FACT-F scale range from 0–4; the range of possible scores is 0–52, with 0 being the worst possible score and 52 the best. Convergent and discriminant validity testing has previously demonstrated a significant positive relationship with other measures of fatigue and a negative correlation with vigor [46]. Cronbach alpha for our sample was 0.95.

#### Perceived stress scale (PSS)

The PSS is a 10 item Likert scale that measures global life stress, by assessing the degree to which experiences are appraised as uncontrollable and unpredictable [47]. Scores can range from 0 to 40, with higher scores indicating greater level of perceived stress. Reliability (stability) is reported as 0.85, with Cronbach alphas ranging from 0.75-0.86 [48]. Cronbach alpha for our sample was 0.84.

#### Functional assessment of cancer therapy – general (FACT-G)

FACT-G is a 27-item instrument that measures quality of life based on four domains: physical well being, social/family well being, emotional well being, and functional well being. For the purposes of this study, the analyses only used the total score, representing a composite of quality of life. FACT-G has established reliability, with a Cronbach alpha of 0.92 and a reported test-retest reliability of 0.93 [46]. The total score range for FACT-G is from 0 to 108, with higher scores indicating greater general well being. Cronbach alpha for our sample was 0.94.

### Immune measures

#### Isolation of peripheral blood mononuclear cells

Blood was collected (between 9 AM and 4 PM) in sterile heparinized tubes and processed immediately. Peripheral blood was overlaid onto Ficoll/Hypaque and centrifuged at 1000 x g for 20 min. The peripheral blood mononuclear cells (PBMC) at the interface were washed twice with Hank's Balanced Salt Solution prior to assessment, as described previously [49].

#### Natural killer cell activity (NKCA)

NKCA was determined using a tumor cell cytotoxicity assay, as previously described [23]. K562 tumor cells, obtained from the American Type Culture Collection, Rockville, MD, maintained in vitro in Corning 25 cm^2^ tissue culture flasks (Corning Glass Works, Corning, NY) in RPMI 1640 (Gibco Laboratories, Grand Island, NY) supplemented with 10% fetal bovine serum (FBS) low LPS; (Gibco Laboratories, Grand Island, NY), 100 units/ml penicillin, 100ug/ml streptomycin (Whittaker M. A. Bioproducts, Walkersville, MD), 0.1 Mm non-essential amino acids and 2 Mm L-glutamine (Gibco Laboratories, Grand Island, NY). For the assay K562 cells were washed once in culture medium, pelleted by centrifugation at 500 x g for 10 min and resuspended in approximately 0.1 ml of culture medium. Then a 100 μci of ^51^Cr (Perkin-Elmer, Warrenville, IL) was added to approximately 1 x 10^7^ cells in a final volume of 0.2 ml. The cells were incubated at 37° Celsius with 5% CO2 for one hour with agitation every 10 min. Subsequently, the cells were washed four times in HBSS, resuspended to 5 x 105 cells/ml in culture medium and 0.01 ml (5 x 10^3^) was aliqouted to each well of a 96 well, round bottom assay plate (Corning Glass Works, Corning, NY). PBMCs and radiolabeled cells were cultured for four hours. Following four hours of incubation, the supernates were removed using a Skatron harvesting press (Skatron Inc., Sterling, VA) and the associated radioactivity was determined using the Cobra II Series Auto Gamma Counting System by Packard Instrument Company (Meriden, CT). Maximum release was obtained by adding 0.05% Novidet P-40 (Sigma Chemical Co., St. Louis, MO). Results are expressed as % cytotoxicity and calculated by the formula below:

(1) $$\%\text{Cytotoxicity}=\frac{\left(\text{experimental DPM}*\right)–\left(\text{minimum DPM}\right)\text{× }100}{\left(\text{maximum DPM}\right)–\left(\text{minimum DPM}\right)}$$

(2) *DPM=disintegrations per minute

All experimental means were calculated from triplicate values. Lytic units (LU) were calculated by a program written by David Coggins (FCRC, Frederick, MD) and reflect the number of cells per 10^7^ effectors required to achieve 20% lysis of the targets.

#### Statistical analysis

Hierarchical linear models (HLM) 6.08 software was used to compute multilevel model of change [50], based on full maximum likelihood estimation. This approach was used to examine intra-individual and inter-individual differences in baseline and trajectories of change over time in fatigue, quality of life, perceived stress, and NKCA. The post radiotherapy time points were anchored with respect to the end of radiation therapy; women in the two groups had different data collection schedules that were accommodated by this statistical approach. Unlike the traditional analysis of variance for repeated measures, HLM treats time as a continuous variable letting each participant have her own data collection schedule (i.e., dependent on type of radiation therapy).

In HLM with longitudinal data, the outcome variables (i.e., fatigue, quality of life, perceived stress, and NKCA) are conceptualized to be nested within individuals and the growth modeling of change in these variables has two levels. At Level 1, the outcome variable is a function of within-person change parameters plus error. At Level 2, outcomes are modeled as a function of predictor variables that vary between participants (i.e., type of radiation treatment and demographic factors), plus an error associated with each individual [50].

HLM analysis was conducted separately for each variable (i.e., fatigue, quality of life, perceived stress, and NKCA) and was performed in two steps. First, potential group effects of radiation treatment were examined without any other variables in the Level 2 models. In the second step, age and use of anti-estrogen endocrine therapy were added to the models to control for potential confounding effects. Time was measured in weeks from the initial visit. The initial pre-treatment visit was coded as zero. Both linear and quadratic trends were examined and goodness-of-fit tests of the deviance between linear and quadratic models were used to assess most appropriate fit. For all models examined, a linear model fit the data better than a quadratic model (p < 0.05).

## Results

### Sample characteristics

Thirty women with early-stage breast cancer were enrolled. Fifteen women received PBRT, Mammosite brachytherapy at dose of 34 Gy 10 fractions/5 days, while fifteen women received WBRT, 3-D conformal techniques at dose of 50 Gy +10 Gy Boost/30 fractions. Means, standard deviations and frequencies of participant demographic characteristics are listed in Table 1. There were no significant differences between the groups with regard to age (t(28) = 0.82, p = 0.42), marital status, race, or adjuvant anti-estrogen endocrine therapy use (*χ*^2^ = 0.05-1.87, p = 0.17-0.82).

**Table 1:** Demographic Characteristics of Participants Based on Type of Radiotherapy

**Demographic Variable**	**Mean (SD)/Percent**	
	WBRT	PBRT
	(n = 15)	(n = 15)
Age (*yrs*)	62.1 (8.35)	64.7(9.03)
Race		
Caucasian	95%	95%
Non-Caucasian	5%	5%
Marital Status		
Married	70%	62%
Divorced/Separated	14%	9%
Widowed	16%	11%
Unknown Status	0%	18%
Anti-Estrogen Endocrine Therapy		
Yes	77%	79%
No	23%	21%

Abbreviations: WBRT = whole breast radiation therapy, PBRT = partial breast radiation therapy.

### Effects of radiation therapy: Inter-individual variation

**Figure 1:**
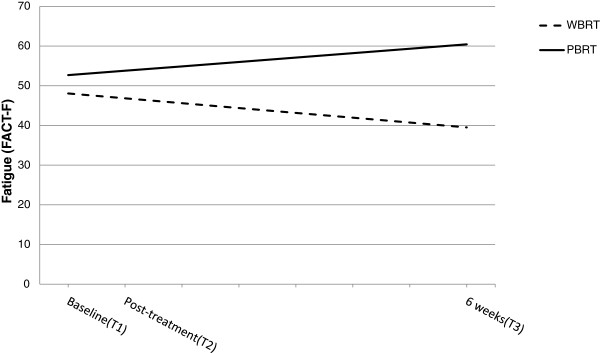
**Influence of type of radiation therapy on trajectories of fatigue. **Graphical representation of the relationship between type of radiotherapy (WBRT = whole breast radiation therapy, PBRT = partial breast radiation therapy) and fatigue (FACT-F scores, score range 0–52) as estimated by the hierarchical linear model. Note higher score indicates less fatigue from the time of the initial assessment (Baseline) to 6 weeks post radiotherapy. Level of fatigue was similar for both groups at the initial assessment. Fatigue increased for women who received WBRT and decreased for women who received PBRT over the 6 weeks post radiation therapy. *b_slope x group_ = 1.11, *p* = 0.04.

**Figure 2:**
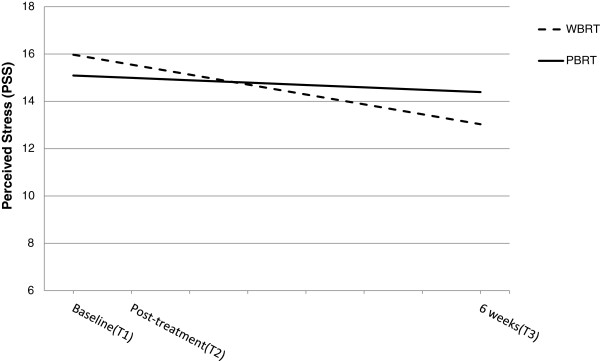
**Influence of type of radiation therapy on trajectories of perceived stress. **Graphical representation of the relationship between type of radiotherapy (WBRT = whole breast radiation therapy, PBRT = partial breast radiation therapy) and perceived stress (PSS scores, score range 0–40) as estimated by the hierarchical linear model from the time of the initial assessment (Baseline) to 6 weeks post radiotherapy. No group differences were found with respect to the level of perceived stress at baseline or as change over time. NS b_slope-intercept_ = −.10 and -.42, *p*-values =0 .78 and 0.31.

**Figure 3:**
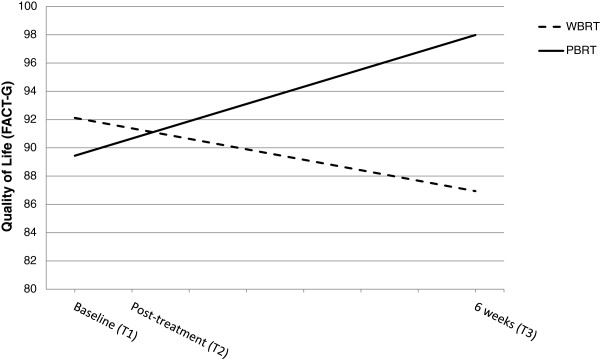
**Influence of type of radiation therapy on trajectories of quality of life. **Graphical representation of the relationship between type of radiotherapy (WBRT = whole breast radiation therapy, PBRT = partial breast radiation therapy) and quality of life (FACT-G scores, score range 0–108) as estimated by the hierarchical linear model from the time of the initial assessment (Baseline) to 6 weeks post radiotherapy. At the initial assessment, groups did not differ with respect to quality of life. However, quality of life declined for women who received WBRT, whereas quality of life increased for women who received PBRT over the 6 weeks post radiation therapy. *b_slope x group_ = 1.11, *p* = 0.05.

**Figure 4:**
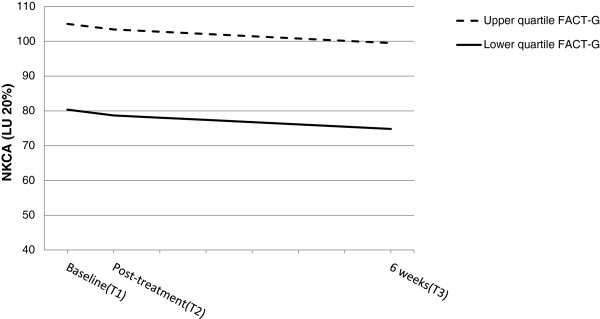
**Effect of quality of life on inter-individual differences in the intercept for NKCA.** Graphical representation of the relationship between quality of life (FACT-G scores) and natural killer cell activity (NKCA) as estimated by the hierarchical linear model from the time of the initial assessment (i.e., Baseline) to 6 weeks post radiotherapy . The slopes for NKCA were based on differences in quality of life levels, calculated as the average upper and lower quartiles. Greater levels of NKCA at baseline and post-treatment were associated with higher quality of life. *b_intercept_ = 1.9, *p* = 0.02.

A longitudinal design was used to compare the effects of WBRT versus PBRT on fatigue, perceived stress, quality of life and NKCA in women with early stage breast cancer. HLM analysis estimated trajectories of outcome variables from the initial assessment prior to radiation therapy through a projected 6-week period of time post radiation therapy. Estimates of fixed and random effects for the final models, including covariates, are presented in Table 2 (summary for all patients). The fixed effects capture systematic inter-individual differences in change trajectory according to the values of the predictors, whereas random effects represent estimated residual variance. Figures 1, 2, 3 and 4 illustrate the effects of the two types of radiation therapy on the trajectories of study outcomes. The mean scores for the outcome variables depicted in the figures are estimated or predicted by the HLM results.

**Table 2:** Hierarchical Linear Models of Quality of Life, Fatigue, Perceived Stress, Natural Killer Cell Activity

**Variable**	**Unstandardized Coefficients (SE)**			
	**Quality of Life (FACT-G)**	**Fatigue (FACT-F)**	**Perceived Stress (PSS)**	**NKCA (Natural Killer Cell Activity)**
Fixed effects:				
Intercept	92.2 (21.5)**	48.1 (4.0)**	16.0 (2.9)	106.9 (38.7)
Time ^*a *^(linear)	-.74 (.46)	-1.22 (.56)*	-.42 (.39)	-5.00 (3.28)
Intercept:				
Group	4.44 (4.59)	4.62 (3.48)	-.88 (2.50)	-25.86 (33.52)
Age	0.08 (.29)	.14 (.22)	-.22 (.16)	1.03 (2.14)
HT	1.25 (5.81)	3.22 (3.39)	-3.19 (3.18)	-7.91 (45.95)
Linear Slope				
Time x Group	1.11 (.56)*	1.11 (.51)*	-.10 (.35)	2.06 (3.04)
Time x Age	.04 (.04)	.04 (.03)	.01 (.02)	-.18 (.08)
Time x Anti-Estrogen Therapy	.40 (.83)	.86 (.61)	.10 (.44)	2.97 (3.08)
Variance Components				
In intercept	116.89***	62.89***	31.37***	6514.53***
In linear slope	1.41**	.48	.10	31.36**

Abbreviations: FACT-G = Functional Assessment of Cancer Therapy – General; FACT-F = Functional Assessment of Cancer Therapy - Fatigue, PSS = Perceived Stress Scale, NKCA = Natural Killer Cell Activity.

^*a*^Time was coded 0 at the time of the diagnosis. *p < 0.05, **p < .01, ***p < 0.001.

### Fatigue

Fatigue was assessed by administering the FACT-F, in which higher scores indicate less fatigue. Fatigue was significantly associated with change over time, such that for women who received PBRT, fatigue was estimated to decrease (b = 1.11, *p* = 0.04); in contrast, for women who received WBRT, fatigue was estimated to increase (b = −1.22, *p* = 0.04) with each additional week (Figure 1). There were no group differences in the initial level of fatigue (i.e., prior to initiation of radiation therapy) (*p* = 0.11). None of the demographic characteristics were associated with reports of fatigue. There was a significant amount of individual variation, as indicated by the variance components of the model, in the initial level of fatigue (*p* < 0.0001), but not in the slope (*p* = 0.17). This suggests that the type of radiation therapy that women received was able to sufficiently explain the amount of variance in the trajectories associated with each participant; however, additional factors (not assessed in the present study) likely contributed to the unexplained variance at the initial status.

### Perceived stress

No group differences in the initial level or the change in the level of perceived stress were observed. As shown in Table 2 and Figure 2, the level of perceived stress remained relatively stable for both WBRT (b = −0.42, *p* = 0.31) and PBRT (b = −0.10, *p* = 0.78). None of the demographic variables were significantly associated with perceived stress. Further, as indicated by the variance components of the model, the type of radiation therapy that women received, along with the covariate variables, sufficiently accounted for the variance in the trajectories associated with each participant (*p* = 0.27); although, at baseline a significant amount of individual variation in perceived stress remained unexplained.

### Quality of life

The FACT–G total summary score was used to assess overall quality of life. HLM analysis revealed no differences in the initial level (i.e., prior to radiation therapy) of quality of life between women who received PBRT or WBRT. However, the trajectories of change over the post-treatment period were different for women who received PBRT as compared to the WBRT group. As demonstrated in Figure 3, a significant linear increase in quality of life was estimated for women receiving PBRT (b = 1.11, *p* = 0.05), whereas quality of life slightly decreased for women receiving WBRT (b = −0.74, *p* = 0.11). The effect of radiation therapy on the change over time in quality of life remained statistically significant after age and use of anti-estrogen endocrine therapy were controlled for in the model. Neither of these covariates were significant predictors of either the intercept (i.e., initial level) nor the linear slope.

### NKCA

No group differences (WBRT versus PBRT) were observed in initial levels of NKCA or in the change in NKCA over time. The demographic characteristics were also not significant predictors of NKCA. Given the heterogeneity of NKCA, an additional model, as described below, was evaluated to further explore the individual change in NKCA over time post-treatment.

### Additional analysis

For the following analysis, data for both groups (WBRT and PBRT) were combined. The model investigated fatigue and quality of life as predictors of the change in NKCA (see Figure 4). Both predictor variables were treated as time-variant variables and were entered simultaneously into the Level 1 model. Results indicated that quality of life, but not fatigue, was significantly associated with change in NKCA levels. For those women who reported better quality of life, a higher level of NKCA was estimated (b = 1.9, *p* = 0.02), which remained elevated post treatment. Both random effects components for the initial level and the linear trend were significant (*p* < 0.001), suggesting that some individual variability remained unexplained. One possibility is that some portion of this variability was due to the variance in blood collection times (i.e., 9 AM to 4 PM); however, this is unlikely because no correlation was observed between time of blood draw and NKCA.

## Discussion

Novel modalities to administer radiation therapy for early stage breast cancer have received increased attention. Partial breast irradiation using brachytherapy delivers radiation to a smaller volume of breast tissue by directing radiation to the tissue adjacent to the excised lesion. Evidence demonstrates that partial breast irradiation results in excellent outcomes with respect to local tumor control, toxicity, and cosmesis at five year follow-up [1-4].

In contrast, a recent SEER database report suggested that PBRT may be inferior to WBRT, with a doubling of recurrence rates in women over 66 years of age [51]. However, this was a retrospective surrogate analysis of insurance data. Moreover, the suitability of patient selection for breast brachytherapy (PBRT) has been called into question [52-54], with one SEER study [54] showing that 65% of women receiving PBRT fell in the unsuitable cautionary group for PBRT based on the American Society of Radiation Oncology guidelines. This may explain the increased local recurrence reported by Smith et al.,[55]. At this time, definitive conclusions regarding local control and survival outcomes for PBRT must await completion of an ongoing national study comparing PBRT to WBRT [5,6].

PBRT may offer other advantages for women undergoing adjuvant radiation therapy for breast cancer. Since WBRT irradiates a greater volume of breast and normal tissues than PBRT, it may lead to greater and more prolonged treatment-associated symptoms than PBRT. For some women the longer duration of daily therapy sessions can be physically and emotionally taxing and PBRT may be better tolerated [14]. Our findings demonstrate that women who receive PBRT, delivered for five days with brachytherapy, exhibit a trajectory of decreasing fatigue, compared to women receiving WBRT, who exhibit worsening fatigue after radiation therapy. These results are consistent with a recent retrospective study, which showed that accelerated (3 weeks) partial breast irradiation resulted in lower maximum fatigue during treatment and lower severe fatigue at treatment completion compared to conventional 6-week whole breast irradiation [14]. Treatment modalities with lower associated fatigue are clinically meaningful, as fatigue is one of the most burdensome symptoms experienced by cancer patients [7]. For most individuals receiving radiation therapy, fatigue subsides to pre-treatment levels within 4–8 weeks after treatment completion [56-58]. Yet, for some cancer survivors, fatigue can become a chronic disabling condition, persisting for months or years after successful cancer treatment [12].

A prior small study evaluated quality of life in women receiving PBRT and showed improvements (change from pre-surgical values) in emotional well being at 1 month post-PBRT, which was followed months later by gains in social/family well-being [43-45]. Yet that study did not compare outcomes of quality of life for women receiving PBRT to that of women receiving WBRT, nor was fatigue evaluated. Our results show baseline levels of quality of life to be good for both groups [59]. However, over time post-radiation quality of life shows an increasing trajectory in quality of life for women receiving PBRT, but a decreasing trajectory of quality of life for women receiving WBRT. Based on established criteria for what is considered a minimally important difference (i.e., >5 point difference for the FACT-G total score), the difference in quality of life between these groups is considered meaningful [60-62].

It is possible that the increasing trajectory in quality of life we observed for women receiving PBRT may be related to lower fatigue, as previous findings confirm that cancer-related fatigue significantly interferes with the course of daily living, diminishing quality of life [10,63]. For women in the present study, baseline levels of fatigue for both groups of women are within the range of general population norms reported for the FACT-F scale [64]. However, post-radiation we observe a decreasing trajectory of fatigue for women who received PBRT, but an increasing trajectory for women who received WBRT. By 6 weeks post-radiation, the lower fatigue for women in the WBRT is at levels within the range reported for non-anemic cancer patients [60]. Further, the increasing difference in fatigue observed across trajectories can be interpreted as meaningful, as the minimal important difference for the FACT-F scale is established to be in the range of 3–4 points [65,66]. A shorter more focused course of radiation therapy that results in lower fatigue and higher quality of life offers clear advantage for women who are unable to tolerate either a mastectomy or a longer course of radiation treatment. This is an important consideration for elderly women with breast cancer [67], as older age was demonstrated to predict higher fatigue during radiation therapy for breast cancer [14].

The underlying factors contributing to the differences we observed in fatigue for women who received WBRT versus those who received PBRT remain unclear. Psychological factors are known to contribute to variation in radiation-associated fatigue severity and duration [15,68]. For women in the present study, perceived stress scores are moderately elevated at baseline [23,48]. By 6-weeks post-radiation, the perceived stress scores return to levels similar to normative levels reported for women without breast cancer [23]. However, we do not observe differences in perceived stress based on type of radiation therapy. Others show that depressive symptoms predict higher fatigue trajectories for women with breast cancer undergoing radiation therapy [15]. Although we did not measure depressive symptoms, it is possible that women receiving WBRT experience more depressive symptoms, increasing risk for higher post-treatment fatigue.

Biological factors may underlie cancer-related fatigue [22,69,70], in that circulating proinflammatory cytokines can signal the brain and engender behavioral symptoms like fatigue and depression [71,72]. Previous reports demonstrate that women with breast cancer exhibit elevated levels and/or production of proinflammatory cytokines [9,23,24], with concomitant fatigue and depressive symptoms [9,70,73,74]. Moreover, a quantitative meta-analysis concluded that fatigue is associated with elevations in circulating levels of IL-6 in cancer patients [73]. Others identify inflammatory processes as potential mediators of radiation-induced fatigue in breast and prostate cancer patients [22]. This may result from exposure to radiation, which triggers inflammatory processes that promote tissue repair [75,76]. WBRT may generate a greater inflammatory response, resulting in more intense and sustained fatigue and lower quality of life.

NK cells conduct immune surveillance against tumors [27,29,32] and breast cancer is responsive to the anti-tumor effects of NK cells [27,30-34]. Studies show that higher NKCA predicts a better prognosis for cancer patients [77-82]. As well, women with breast cancer who report more behavioral symptom distress exhibit lower NKCA [23-25], which may be mediated by elevations in stress hormones [80,83-85]. Our results did not reveal differences in NKCA based on type of breast radiation therapy. This may be related to the lack of observed differences in perceived stress between the two treatment groups. However, *post hoc* analysis revealed that the perception of better quality of life predicted higher NKCA, post-treatment, for both groups of women (i.e., WBRT and PBRT). We previously showed that a mindfulness based stress-reduction program for women undergoing breast cancer treatment improved quality of life, as well as reduced cortisol levels and increased NKCA restoration after cancer treatment [24]. It is possible that the perception of better quality of life during cancer treatment, as observed in this study, may reduce endocrine stress signals, resulting higher NKCA [69,85].

## Conclusions

In conclusion, these results show that PBRT resulted in lower radiation-associated fatigue and higher quality of life after radiation therapy compared to WBRT. Although this pilot study is limited by the small sample size and the non-randomization of subjects to treatment group, the results identify advantages for choosing PBRT for treatment of breast cancer. Women who begin radiation therapy after adjuvant chemotherapy, women with advanced age, and women with pre-existing co-morbidities are patient subgroups at greater risk for radiation-associated fatigue and poor quality of life; which can interrupt cancer treatment and predispose to poor health outcomes. Thus, these women may benefit from PBRT. Moreover, the findings also demonstrate that better quality of life predicted higher NKCA against tumor targets, emphasizing the importance of fostering good quality of life for women during radiation therapy. This is clinically relevant, as after surgery and during adjuvant treatment patients are at risk for post-surgical tumor dissemination and NKCA is more effective in halting nascent tumor cell seeding when tumor burden is low [35,36,38]. Thus, the results of this investigation provide evidence to assist clinical decision-making regarding approaches for adjuvant radiation therapy after breast conservation surgery.

## Abbreviations

WBRT, whole breast radiation therapy; PBRT, partial breast radiation therapy; NKCA, natural killer cell activity; BCT, breast conservation treatment; NK, natural killer; PSS, perceived stressor scale; PBMC, peripheral blood mononuclear cells; DPM, disintegrations per minute; HLM, Hierarchical linear models; SE, standard error of the mean; SD, standard deviation.

## Competing interests

The authors KA, DT, PL, LM, HM and LJ declare that they have no competing interests either financial or non-financial.

## Authors’ contributions

KA, LJ, HM originally conceived and designed the study and directed the acquisition, analysis and interpretation of data. KA also oversaw radiation oncology clinical aspects of the study and drafted and participated in the completion of the manuscript. LJ also advised on the behavioural measures, recruitment of subjects, and participated in the completion of the manuscript. HM also advised on immune measures and participated in the completion of the manuscript. PL contributed to the design and acquisition of data. LM participated in the recruitment of subjects and the acquisition of patient data. DT implemented the statistical analysis, drafted the results section and figures, and participated in the interpretation of data. All authors read and approved the final manuscript.

## Pre-publication history

The pre-publication history for this paper can be accessed here:

http://www.biomedcentral.com/1471-2407/12/251/prepub
